# The Atypical Guanylate Kinase MoGuk2 Plays Important Roles in Asexual/Sexual Development, Conidial Septation, and Pathogenicity in the Rice Blast Fungus

**DOI:** 10.3389/fmicb.2017.02467

**Published:** 2017-12-11

**Authors:** Xingjia Cai, Xi Zhang, Xinrui Li, Muxing Liu, Xinyu Liu, Xiaoli Wang, Haifeng Zhang, Xiaobo Zheng, Zhengguang Zhang

**Affiliations:** Department of Plant Pathology, College of Plant Protection, Nanjing Agricultural University, and Key Laboratory of Integrated Management of Crop Diseases and Pests, Ministry of Education, Nanjing, China

**Keywords:** guanylate kinase, GTP biosynthesis, conidiogenesis, appressorial formation, infectious growth

## Abstract

Guanylate kinases (GKs), which convert guanosine monophosphate into guanosine diphosphate (GDP), are important for growth and mannose outer chain elongation of cell wall N-linked glycoproteins in yeast. Here, we identified the ortholog of *Saccharomyces cerevisiae* GK Guk1, named MoGuk1 and a novel family of fungal GKs MoGuk2 in the rice blast fungus *Magnaporthe oryzae*. MoGuk1 contains 242 aa with an C-terminal GuKc domain that very similar to yeast Guk1. MoGuk2 contains 810 amino acids with a C-terminal GuKc domain and an additional N-terminal efThoc1 domain. Expression of either MoGuk1 or MoGuk2 in heterozygote yeast *guk1* mutant could increase its GDP level. To investigate the biological role of MoGuk1 and MoGuk2 in *M. oryzae*, the gene replacement vectors were constructed. We obtained the Δ*Moguk2* but not Δ*Moguk1* mutant by screening over 1,000 transformants, indicating MoGuk1 might be essential for *M. oryzae*. The Δ*Moguk2* mutant showed weak reductions in vegetative growth, conidial germination, appressorial formation, and appressorial turgor, and showed significant reductions in sporulation and pathogenicity. Moreover, the Δ*Moguk2* mutant failed to produce perithecia and was sensitive to neomycin and a mixture of neomycin-tunicamycin. Exogenous GDP and ATP partially rescued the defects in conidial germination, appressorial formation, and infectious growth of the mutant. Further analysis revealed that intracellular GDP and GTP level was decreased, and GMP level was increased in the mutant, suggesting that MoGuk2 exhibits enzymatic activity. Structural analysis proved that the efThoc1, GuKc, and P-loop domains are essential for the full function of MoGuk2. Taken together, our data suggest that the guanylate kinase MoGuk2 is involved in the *de novo* GTP biosynthesis pathway and is important for infection-related morphogenesis in the rice blast fungus.

## Introduction

Rice blast, caused by *Magnaporthe oryzae*, is one of the most devastating rice diseases worldwide (Yan and Talbot, 2016; Zhang et al., 2016). Outbreaks of rice blast are a constant threat to global rice production and could result in an annual loss of rice capable of feeding more than half of the global population (Zeigler, 1998; Talbot, 2003; Zhang et al., 2016). The control of rice blast is problematic because of the volatility of rice blast physiological races (Zeigler et al., 1994). An improved understanding of the developmental and pathogenic molecular mechanisms of the fungus will facilitate research into targeted fungicides and disease management (Wilson and Talbot, 2009). A key step in the disease cycle of *M. oryzae* is the formation of a highly specialized infection structure known as an appressorium, which differentiates from the asexual spores (Saunders et al., 2010; Oses-Ruiz et al., 2017). Following maturation, enormous turgor pressure is generated in the appressorium, which forces the penetration peg to physically puncture the plant cuticle (Howard et al., 1991; Dejong et al., 1997). After invading host cells, infectious hyphae (IH) differentiate and rapidly extend into plant cells, with a new disease cycle reinitiating in 5–7 days (Foster et al., 2017). To complete the infection cycle, *M. oryzae* evolved genetic regulatory systems that allow it to respond to available nutrients in the plant host and also encounter nitrogen-starved environments at the start of the infection cycle (Snoeijers et al., 2000; Wilson et al., 2007; Fernandez et al., 2014). The regulatory systems include nitrogen metabolite repression (NMR) and carbon catabolite repression (CCR) to ensure the use of preferred sources of nitrogen (ammonium and L-glutamine) and carbon (glucose), respectively (Wilson et al., 2007, 2012; Wilson and Talbot, 2009; Fernandez et al., 2012). Many regulators (such as Nut1, Npr1/2, Nir1, Rbp35, Snf1, Tps1, Nmr1-3, and Mdt1) involved in these systems were characterized to be important for appressorial formation and penetration, as well as infection in *M. oryzae* (Froeliger and Carpenter, 1996; Lau and Hamer, 1996; Wilson et al., 2007, 2010, 2012; Yi et al., 2008; Franceschetti et al., 2011; Fernandez et al., 2012).

**Figure 1:**
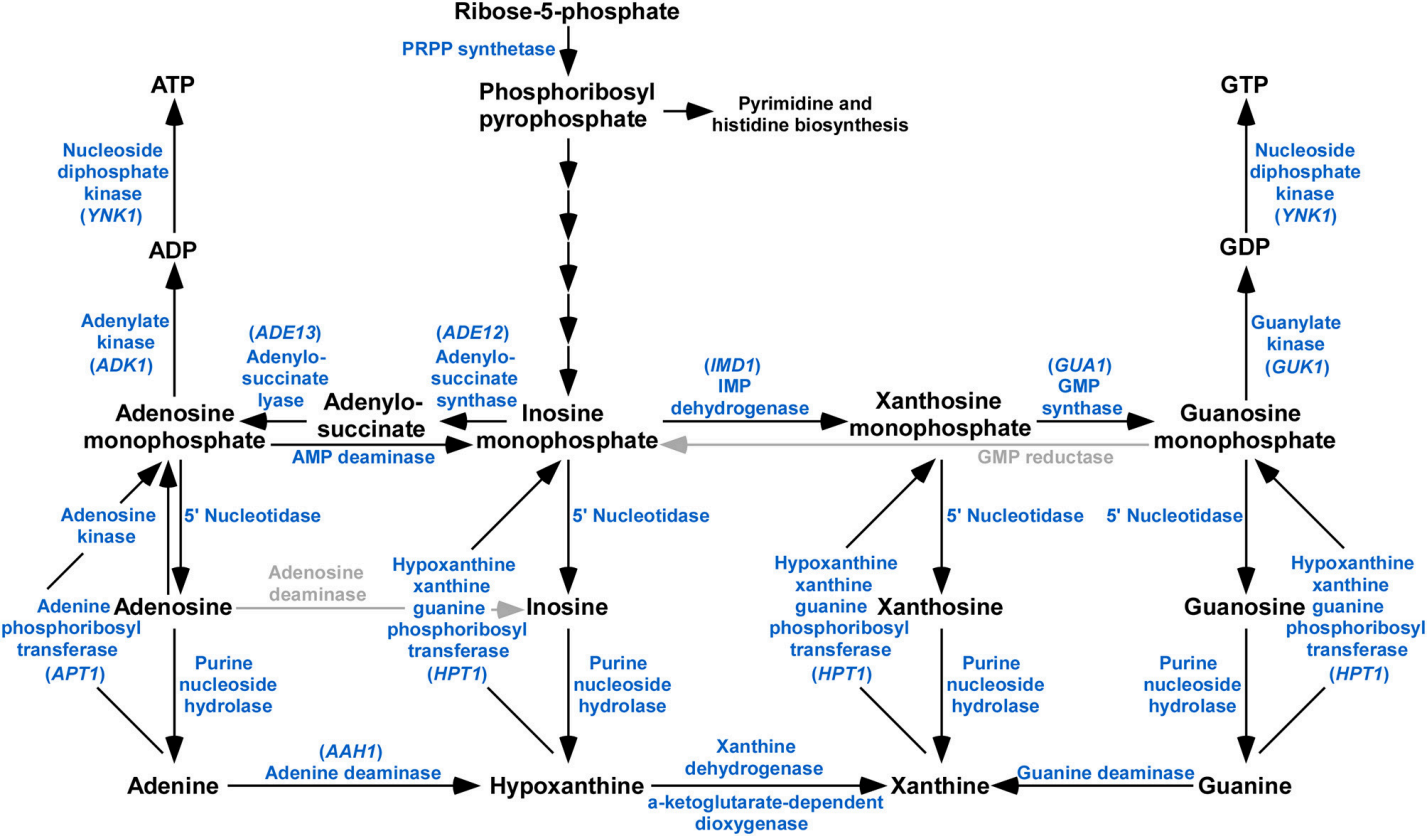
Schematic representation of purine metabolism pathway identified in *M. oryzae*. BLASTp analysis using *S. cerevisiae* and *C. neoformans* orthologs reveals that majority of the components of the purine biosynthetic pathway are present in the genome of *M. oryzae*. Enzymes or activities missing are grayed.

The purine metabolic pathway provides the cell with adenosine triphosphate (ATP) and guanosine triphosphat (GTP), which are critical to cellular processes (Elion, 1989). Purine nucleotides are synthesized by both *de novo* and salvage pathways in organisms (Nyhan, 2005). The biochemistry of *de novo* purine nucleotide biosynthesis and salvage is a broadly conserved and well-understood component of cellular metabolism (Beck et al., 2003). The *de novo* purine nucleotide biosynthesis pathway is the pre-dominant method for purine nucleotide generation, whereby phosphoribosyl pyrophosphate (PRPP) is the initial substrate and, via a series of enzymatic reactions, and formed the purine nucleotide inosine monophosphate (IMP) (Liechti and Goldberg, 2012). IMP is the central product of both *de novo* and salvage pathways and central to the interconversion to adenine and guanine nucleotides (Hedstrom, 2009; Sunohara et al., 2013). IMP could be utilized to generate xanthomine monophosphate (XMP) and then to guanine monophosphate (GMP) by the dual reactions of IMP dehydrogenase and GMP synthase (Sunohara et al., 2013), or to produce adenosine monophosphate (AMP) catalyzed by the enzymes of adenylosuccinate synthase and adenylosuccinate lyase (Liechti and Goldberg, 2012; Morrow et al., 2012; Guibinga et al., 2013). The other pathway for purine nucleotide biosynthesis is the purine salvage pathway (Figure 1). The process of purine salvage can generate GMP from guanine and AMP from adenine directly, or generate IMP or XMP from hypoxanthine and xanthine indirectly. These nucleotides can then be fed into the nucleotide biosynthesis pathway, where they can then be used to generate GMP or AMP. In addition, based on the established pathway, enzymes such as adenine deaminase, and guanine deaminase allow the cycling of nucleotides bypasses the *de novo* and salvage pathways, thereby allowing for various purine bases to be used for generating both GMP and AMP (Pettersson et al., 2007).

Guanylate kinase (GK) is an essential enzyme that catalyzes the transfer of a phosphate from ATP to GMP (Sekulic et al., 2002). The enzyme is indispensable for converting GMP to GDP and therefore synthesis of GTP (Li et al., 1996). Plants have two distinct types of GKs, which are essential for growth and development (Nomura et al., 2014). Both yeast and humans have a single essential GK involved in GDP biosynthesis (Konrad, 1992; Jain et al., 2016). Here, we identified and characterized the GK MoGuk1 and MoGuk2 in the phytopathogen *M. oryzae*. We proved that MoGuk1 likely was essential for *M. oryzae*, and MoGuk2 is involved in the *de novo* GTP biosynthesis pathway and is important for development and virulence in the rice blast fungus.

## Materials and methods

### Strains and culture conditions

The wild type Guy11 (ATCC201236) and mutant strains of *M. oryzae* were cultured on complete medium (CM) agar plates at 28°C. Fungal mycelia were cultured and harvested from 2-day-old liquid CM medium by filtration through one layer of Miracloth, and used for genomic DNA and total RNA extraction. Protoplasts preparation and *M. oryzae* transformation was performed as described previously (Sweigard et al., 1992). For vegetative growth, mycelial plugs (3 × 3 mm) were placed onto CM, minimal medium (MM), oatmeal medium (OM), and straw decoction and corn (SDC) medium and cultured at 28°C for 7 days (Talbot et al., 1993; Song et al., 2010; Zhang et al., 2010, 2014). Each experiment was performed in triplicate and repeated three times.

### Construction of *MoGUK1* and *MoGUK2* null mutant, domain deletion, and complementation vectors

The gene deletion construct was generated according to the homologous recombination principle as described (Tang et al., 2015). In brief, a flanking sequence fragment of *MoGUK1* and *MoGUK2* (1-kb upstream and 1-kb downstream) was amplified from *M. oryzae* genomic DNA and cloned into the pCX62 vector to flank the hygromycin phosphotransferase cassette (*HPH*). The resulting pCX62::*MoGUK1*::*HPH* and pCX62::*MoGUK2*::*HPH* vectors were confirmed by sequence analysis and used for protoplast transformation. For domain deletion and full length complemented constructs generation, the entire *MoGUK2* gene or domain deletion fragments and its native promoter region (1,500 bp upstream from the starting codon) was amplified by PCR and cloned into the pYF11(bleomycin resistance) vector using a yeast gap repair approach, respectively (Bruno et al., 2004). The final constructs were sequenced and transformed into the Δ*Moguk2* mutant to obtain the full length complemented transformant and domain deletion transformants. The primers used in this section are listed in Table S1.

### Conidiation, sexual reproduction, appressorial formation, and turgor assays

For conidial development on glass slides assay, 5 × 5 mm mycelial plugs were cut from the indicated strains cultured on SDC plates for 7 days, and placed on glass slides to induce conidiation under black light for 20 h, and observed under a light microscope. For conidial quantification assay, the indicated strains were inoculated on SDC plates to promote conidiation as described (Zhang et al., 2009). Conidia were collected in distilled water and filtered through with one layer Miracloth (Millipore, USA). Conidial number was counted with a hemocytometer under a microscope. For sexual reproduction, plugs of the Δ*Moguk2* mutant and the wild type Guy11 (*MAT1-2*) and the mating partner strain TH3 (*MAT1-1*) were point-inoculated 3 cm apart on oatmeal agar medium and cultured at 20°C with continuous light for 3–4 weeks as described (Zhang et al., 2011). Mature perithecia were crushed to examine the asci and ascospores and photographed. Appressorial formation was performed by placing conidial suspensions (1 × 10^4^ spores/ml) on cover glass (Fisher-brand, UK) (hydrophobic surface) and cultured at 28°C. Conidial germination and appressorial formation was examined under a microscope by time course. Appressorium turgor was measured by incipient cytorrhysis (cell collapse) assay using a 1–4 molar concentration of glycerol solution as described previously (Howard et al., 1991). All experiments were performed in triplicate and repeated three times.

### Plant infection and infectious hyphal growth assays

Plant infection was conducted on 2-week old rice seedlings and 1-week old detached barley leaves as described previously (Zhang et al., 2011; Li et al., 2016). Diseased leaves were observed daily and photographed at 5 (barley) or 7 (rice) days post-inoculation (dpi). For infectious hyphal growth assay, the rice leaf sheaths were inoculated with 100 μl conidial suspensions (1 × 10^5^ spores/ml), and incubated in a humid chamber at 28°C. Infectious hyphal growth was observed at 30 or 40 h post-inoculation (hpi) under a microscope.

### GDP, GTP, and GMP extraction and HPLC analysis

All of the strains were cultured on CM medium at 28°C, cut into 1 × 1 mm square and cultured in liquid CM for 2 days. Filtering to collect mycelium and quickly ground into powder. Every 1 mg mycelium mixed with 20 μl 6% TCA solution, turbulenced. Samples were centrifuged (4,000 rpm, 15 min), the top layer were collected and washed with five times the volume of anhydrous ether twice. The pellet was collect for HPLC (High Performance Liquid Chromatography). HPLC analysis was done with a programmable Aglient Technology zorbax 1200 series liquid chromatograph. The solvent system consisted of methanol (90%): water (10%), at a flow rate of 1 ml/min. Solution (GDP, GTP, and GMP) was eluted through the column (SB-C18, 5 μl, 4.6 × 250 mm) and detected at 259 nm UV. Each sample through the column in turn and detected the peak value with the same time as the standard.

### Yeast complementation assay

The full length cDNA of *MoGUK1* and *MoGUK2* was amplified using primers (Table S1), and cloned into the yeast expression vector pYES2 plasmid. The resulting plasmids were verified by sequencing and transformed into yeast mutant strain (BY4743; MAT a/alpha; hisΔ1/his3Δ1; leu2Δ0/leu2Δ0; lys2Δ0/LYS2; MET15/met 15Δ0; uraΔ0/uraΔ0; YDR454c::kanMX4/YDR454c) which bought from EUROSCARF. Yeast protein extraction was conducted as follows: Cells were cultured in liquid SDgal media to mid log phase and centrifuged at 5,000 rpm, 4°C, 5 min; Washed once in 1 ml cold 0.85% NaCl; Re-suspended the cells in 200 μl lysis buffer, added 100 μl of glass beads, and vortex 5 min at 4°C; then centrifuged at 4°C, 13,000 rpm for 10 min and transferred supernatant to new tubes. The samples were used immediately for HPLC analysis as performed above.

### Nucleic acid manipulation, quantitative RT-PCR, and southern blot assays

The *MoGUK2* gene probe and *HPH* probe were amplified with the primer pairs, respectively. Probe labeling, hybridization, and detection were performed with a DIG High Prime DNA Labeling and Detection Starter Kit (Roche). Total RNA was isolated from frozen fungal mycelia using an RNA extraction kit (TaKaRa, China). Total RNA was pretreated with DNase I and was then reverse transcribed (TaKaRa, China). Quantitative RT-PCR was performed on an ABI 7500 real-time PCR system (Applied Biosystems, USA) according to the manufacturer's instructions. The stable expression *ACTIN* gene (MGG_03982) was used as internal control. All primers used in this section are listed in Table S1.

## Results

### Identification and domain prediction of MoGUK proteins in *M. oryzae*

**Figure 2:**
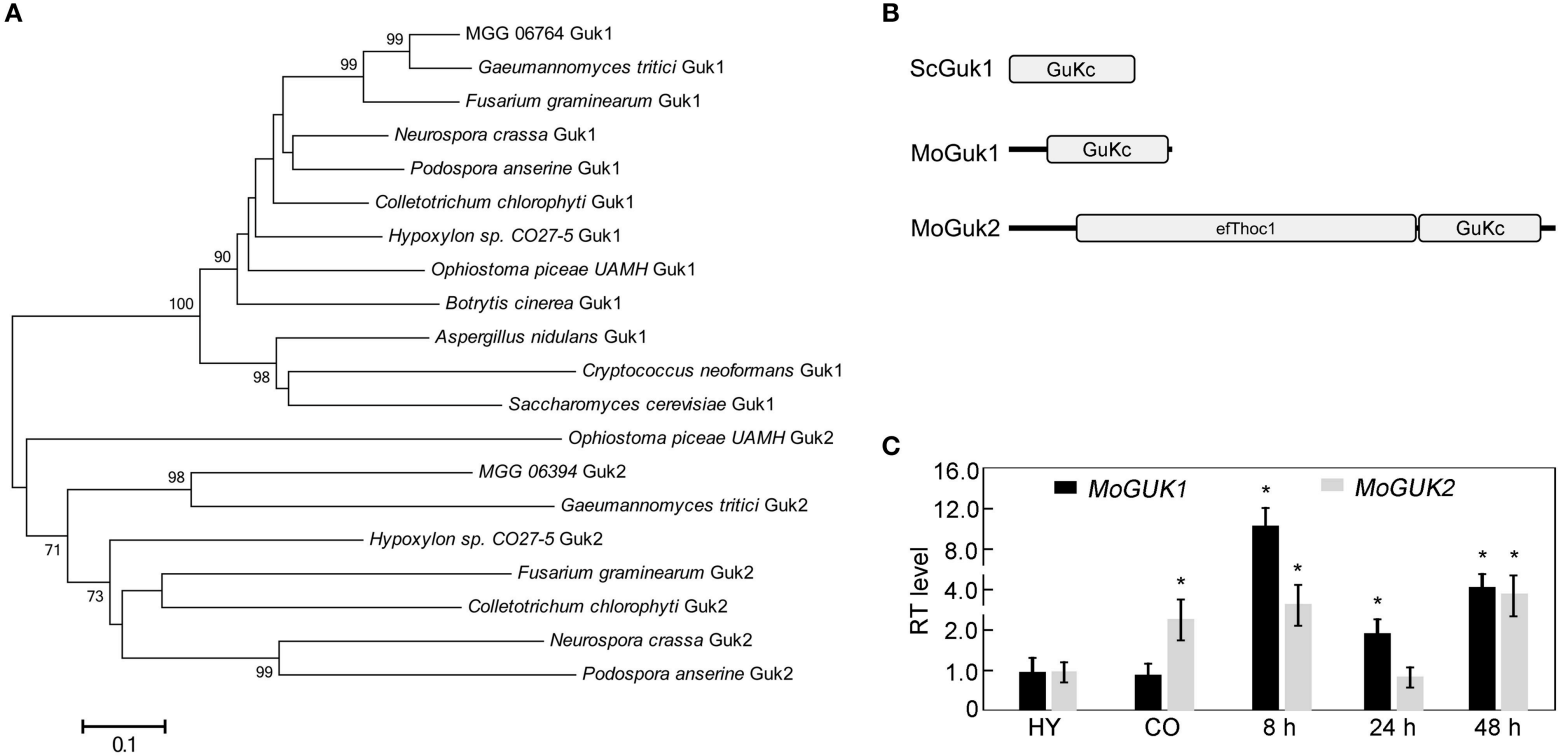
Phylogenetic analysis of Guk proteins from different organisms. **(A)** The homologs of ScGuk1 in *M. oryzae* were identified using blastp program and 1e-40 as an *e*-value cut-off. To construct the tree, homolog sequences retrieved from GuKc domains of MoGuk1 and MoGuk2 families in other indicated species were identified using blastp program default parameters. The tree was constructed using MEGA 5, with the Neighbor-Joining method and default parameters, bootstrap values >65 were displayed beside the braches. **(B)** The GuKc domains were predicted using SMART server (http://smart.embl-heidelberg.de). **(C)** Quantitative RT-PCR analysis the expression of *MoGUK1* and *MoGUK2*. Total RNA was extracted from mycelia (MY), conidia (CO), and infectious hyphae of the wild type Guy11. Error bars represent standard deviation and asterisks represent significant differences at *P* < 0.01.

*S. cerevisiae* ScGuk1 protein sequence was used to search the *M. oryze* genome database using blastp (1e^−40^ as an *e*-value cut-off) in FungiDB (http://fungidb.org), and yield the ortholog MoGuk1 (MGG_06764) and a protein with similarity to ScGuk1, named MoGuk2 (MGG_06394). Phylogenetic analysis for the homologs of MoGuk1 and MoGuk2 in some fungal species indicated that MoGuk1, as well as ScGuk1, formed a conserved group that contained one ortholog in each analyzed species; while MoGuk2 orthologs were only found in a few species, but also formed a conserved group that different from Guk1 group, indicating MoGuk2 is a novel family of GKs in *M. oryzae* (Figure 2A).

Sequence analysis revealed that *MoGUK1* comprises 845 base pairs (bp) separated by one intron, encoding a 242-amino-acid (aa) protein with a Guanylate_kin domain (PFAM PF00625) that catalyzes ATP-dependent phosphorylation of GMP to GDP, which is similar to ScGuk1. *MoGUK2* comprises 2,646 bp separated by three introns, encoding an 810 aa protein with a C-terminal Guanylate_kin domain, and an additional N-terminal efThoc1 domain (PFAM PF11957), which is required for transcription elongation through genes containing tandemly repeated DNA sequences and is also involved in mRNA export from nucleus (Figure 2B). Protein sequence alignment exhibited that GuKc domain showed high identities among MoGuk1, MoGuk2, and their orthologs, therefore driven a question that whether the Guk1 and Guk2 groups proteins had similar functions (Figure S1). To test the hypothesis, we constructed the yeast expression vectors pYES2-*MoGUK1* and pYES2-*MoGUK2* and introduced into the diploid yeast strain since *GUK1* is an essential gene in yeast. The result showed that intracellular GDP level of the yeast strain expressed *MoGUK1* (3.54 ± 0.12) or *MoGUK2* (6.72 ± 0.06) was increased compared to the strain expressed empty vector (2.92 ± 0.14), indicating both MoGuk1 and MoGuk2 have GK activity like yeast Guk1. Expression profile analysis revealed that the transcription level of both *MoGUK1* and *MoGUK2* was increased at the early infection stage in comparison to the mycelial stage (Figure 2C). *MoGUK2* also showed high transcription level in conidial stage (Figure 2C). These results implicated that *MoGUK1* and *MoGUK2* plays potential roles in the development and infection of *M. oryzae*.

### MoGuk2 is involved in vegetative growth

To investigate the role of MoGuk1 and MoGuk2 in *M. oryzae*, gene knock-out constructs pCX62::*MoGUK1 U&D*::*HPH* and pCX62::*MoGUK2 U&D*::*HPH* was generated by replacing *MoGUK1* or *MoGUK2* with a hygromycin phosphotransferase cassette (*HPH*), respectively (Figure S2A). The final constructs were transformed into protoplasts of the wild type strain, Guy11. The resulting hygromycin-resistant transformants were first screened by PCR. However, we failed to get a *MoGUK1* null mutant by screening over 1,000 transformants, indicating *MoGUK1* might be an essential gene in *M. oryzae*. Fortunately, four Δ*Moguk2* mutants were obtained from 210 transformants, which further confirmed by Southern blot analysis (Figure S2B). The complemented transformant Δ*Moguk2/MoGUK2* was generated by re-introducing the wild type *MoGUK2* gene into the Δ*Moguk2* mutant.

**Figure 3:**
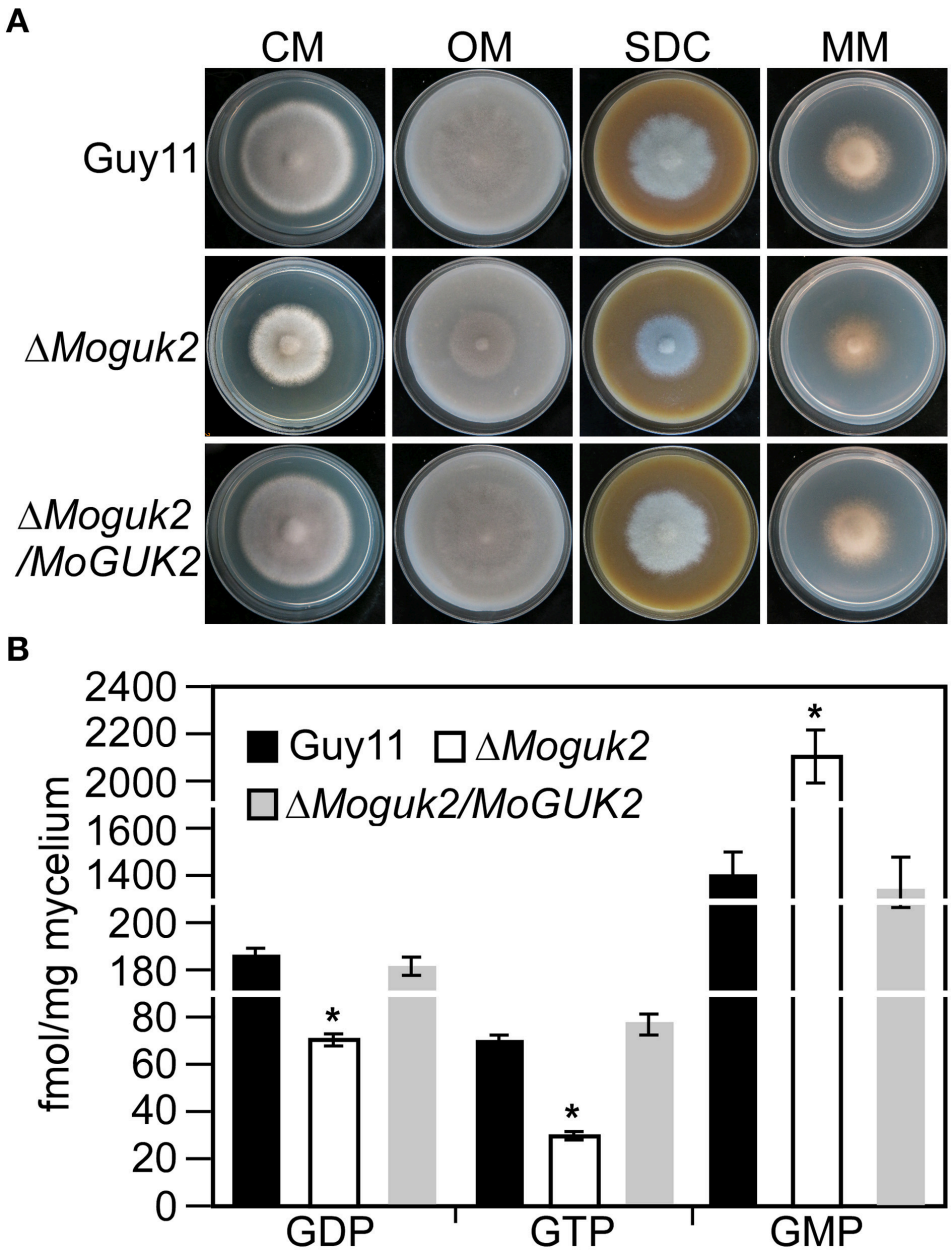
The Δ*Moguk2* mutant decreased vegetative growth and intracellular GDP, GTP level, and increased GMP level. **(A)** Colony morphology was examined on CM, SDC, OM, and MM ager plates, and photographed after culture at 28°C in darkness for 7 days. **(B)** Intracellular GDP, GTP, and GMP measurement. Error bars are standard deviations and asterisks represent significant differences at *P* < 0.01.

We first analyzed the role of MoGuk2 in vegetative growth. Wild type Guy11, Δ*Moguk2* mutant and Δ*Moguk2/MoGUK2* were inoculated onto complete medium (CM), oatmeal medium (OM), straw decoction and corn medium (SDC), and minimal medium (MM) agar plates for 7 days. The mutant displayed no difference in colony morphology in comparison with the wild type or complemented transformant (Figure 3A); however, the growth rate of the Δ*Moguk2* mutant was decreased on these four media. The percentage of reduction in growth was 28.0, 33.3, 27.4, and 18.3% in CM, OM, SDC, and MM, respectively (Table 1). Therefore, MoGuk2 plays a role in vegetative growth of the rice blast fungus.

**Table 1:** Vegetative growth and response to neomycin and tunicamycin in *M. oryzae* strains.

**Strain**	**Growth rate (mm day**^**−1**^**)**^a^				**Growth rate (%)**^b^	
	**CM**	**OM**	**SDC**	**MM**	**3 μg/ml Neomycin**	**Neomycin (3 μg/ml)-tunicamycin (3 μg/ml)**
Guy11	7.5 ± 0.1	7.2 ± 0.1	6.2 ± 0.1	6.0 ± 0.1	98.7 ± 0.2	83.9 ± 0.0
Δ*Moguk2*	5.4 ± 0.0^*^	4.8 ± 0.1^*^	4.5 ± 0.1^*^	4.9 ± 0.0^*^	88.7 ± 1.5^*^	74.1 ± 3.2^*^
Δ*Moguk2MoGUK2*	7.3 ± 0.1	6.9 ± 0.1	5.9 ± 0.1	5.7 ± 0.0	98.2 ± 0.4	83.2 ± 0.6

a *Mean and standard deviation were calculated with results from three replicates. Asterisk represents significant differences at P < 0.01*.

b *The growth rate was determined 7 days after incubation at 28°C by plotting the percentage of colonies in the presence of various drugs against regular CM*.

### Intracellular GDP and GTP level was decreased, and GMP was increased in the Δ*Moguk2* mutant

Guk1 is an enzyme that indispensable for converting GMP to GDP and therefore synthesis of GTP (Li et al., 1996). To investigate whether MoGuk2 have the similar role and indeed involving in GTP biosynthesis pathway, we measured the intracellular GDP, GTP, and GMP levels in the Δ*Moguk2* mutant. The results showed that GDP and GTP level was significantly decreased in the Δ*Moguk2* mutant compared to the wild type Guy11, and decreased 60.1 and 63.7%, respectively. In contrast, intracellular GMP was remarkably increased in the mutant, and increased 34.0% compared to the wild type (Figure 3B). These results indicated that MoGuk2 plays a role in converting GMP to GDP and therefore synthesis of GTP in *M. oryzae*.

### MoGuk2 is important for asexual and sexual development

**Figure 4:**
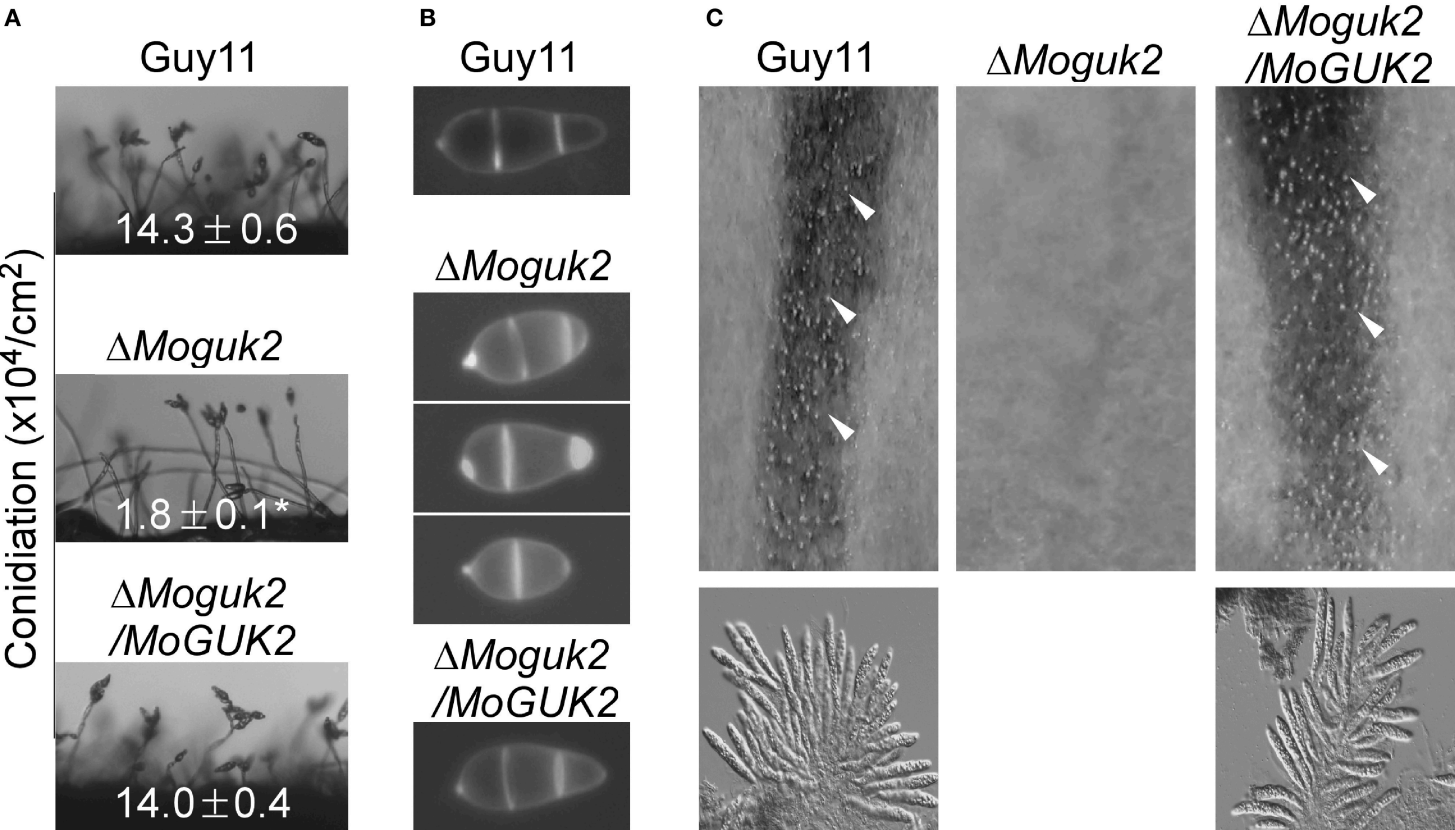
The Δ*Moguk2* mutant showed defects in asexual and sexual development. **(A)** Conidial development was examined under a light microscope 20 h after induction on glass slides. ±*SD* was calculated from three repeated and asterisks represent significant differences at *P* < 0.01. **(B)** Conidial morphology of the Δ*Moguk2* mutant. **(C)** Perithecia and asci formed by the indicated strains were photographed 3 weeks after inoculation. White arrows indicate peritheria.

Conidiation is one of the key steps in the disease cycle of *M. oryzae* (Lee et al., 2006). Therefore, the role of MoGuk2 in conidial production was examined. The three strains were inoculated onto SDC plates to promote conidiation. The Δ*Moguk2* mutant formed normal conidiophores but with fewer conidia compared with the wild type and complemented transformant. The conidial number was decreased 87.4% in the mutant (Figure 4A). We next quantified the numbers of conidia produced by the indicated strains cultured for 10 days on SDC plates. Consistent with the above mentioned result, conidial production by the Δ*Moguk2* mutant was significantly decreased to 12.7% of that by the wild type. In addition, the conidial morphology of the mutant was altered; 53.0% of conidia showed an abnormal morphology with one or two septa, compared with 98.0% normal conidia with two septa in the wild type strain (Figure 4B). Regarding sexual development, the ability of the Δ*Moguk2* mutant to produce perithecia was completely abolished. No perithecia were observed in the mutant, while numerus mature perithecia and asci were observed in the wild type and complemented transformant (Figure 4C). The results above suggest that MoGuk2 plays an important role in conidiogenesis and is essential for sexual reproduction.

### MoGuk2 is indispensable for full virulence of *M. oryzae*

**Figure 5:**
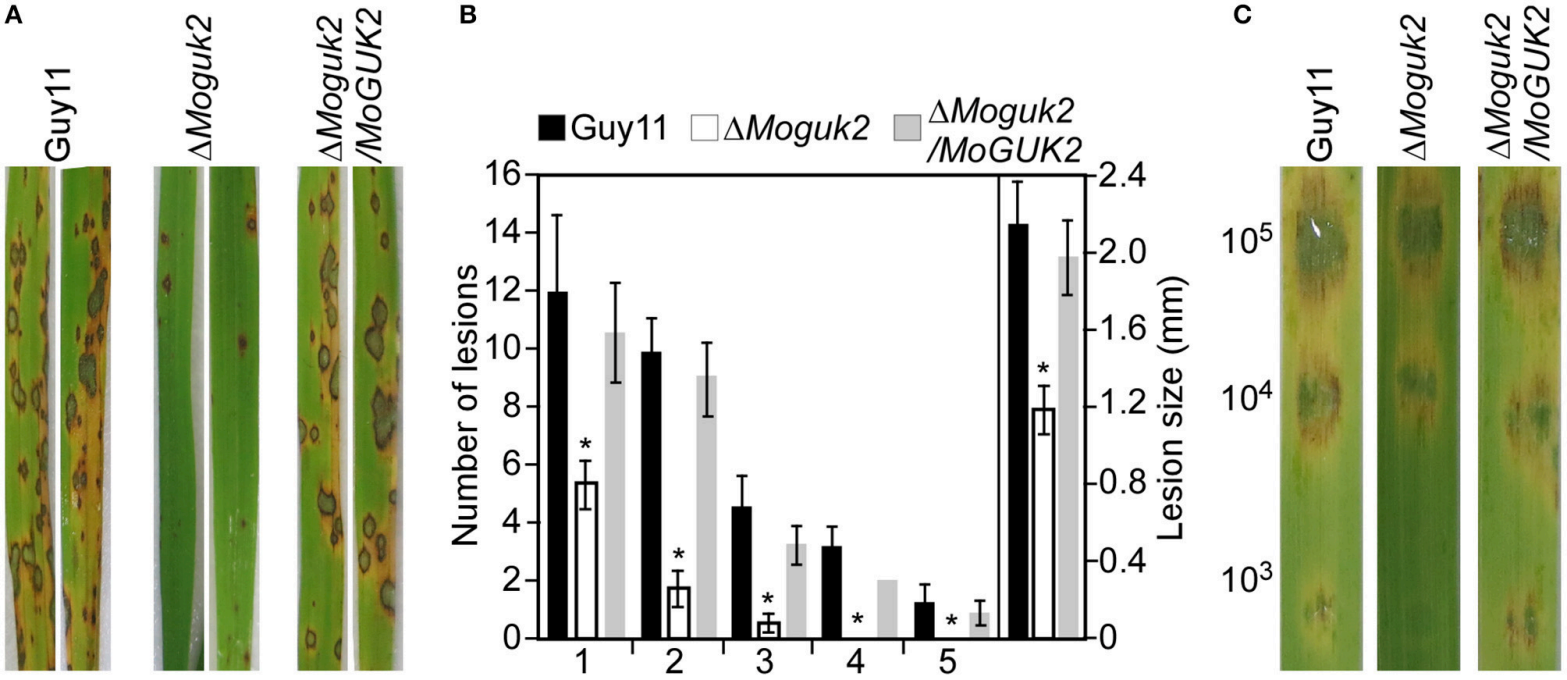
The Δ*Moguk2* mutant attenuates virulence. **(A)** Conidial suspensions (5 × 10^4^ spores/ml) from the wild type, the Δ*Moguk2* mutant and the complemented transformant were sprayed onto 2-week-old rice seedlings, and photographed at 7 days post-inoculation (dpi). **(B)** Statistical analysis of the number of lesions in each type, and the average lesion size of the indicated strains. Lesions on 10 diseased rice leaves were counted per replicate, and the experiment was repeated three times. Error bars are standard deviations and asterisks represent significant differences at *P* < 0.01. **(C)** Conidial suspensions (1 × 10^5^, 1 × 10^4^, and 1 × 10^3^ spores/ml) from the indicated strains were dropped onto 1-week-old detached barley leaves, and photographed at 5 dpi.

To evaluate the role of MoGuk2 in pathogenicity, conidial suspensions prepared from Guy11, the Δ*Moguk2* mutant and the complemented transformant were sprayed onto 2-week-old rice seedlings. At 7 days after inoculation, the mutant caused few small lesions on rice leaves in comparison with the numerous typical lesions caused by the wild type and complemented transformant (Figure 5A). The lesion number was decreased 73.9% compared to the wild type (Figure 5B). Disease severity was also quantified by a lesion-type scoring assay (Valent et al., 1991). The number of lesions of types 1–3 produced by the Δ*Moguk2* mutant was markedly decreased compared with the wild type; moreover, the mutant produced no type 4 or 5 lesions. The average lesion size of the mutant was decreased 45.5% compared to the wild type Guy11 (Figure 5B). Further detached barley infection assays showed similar results; i.e., the mutant was reduced in virulence, and the average lesion size was reduced 26.1% compared to the wild type in detached barley leaf assay (Figure 5C).

### MoGuk2 plays roles in conidial germination, appressorial formation, and turgor generation

**Figure 6:**
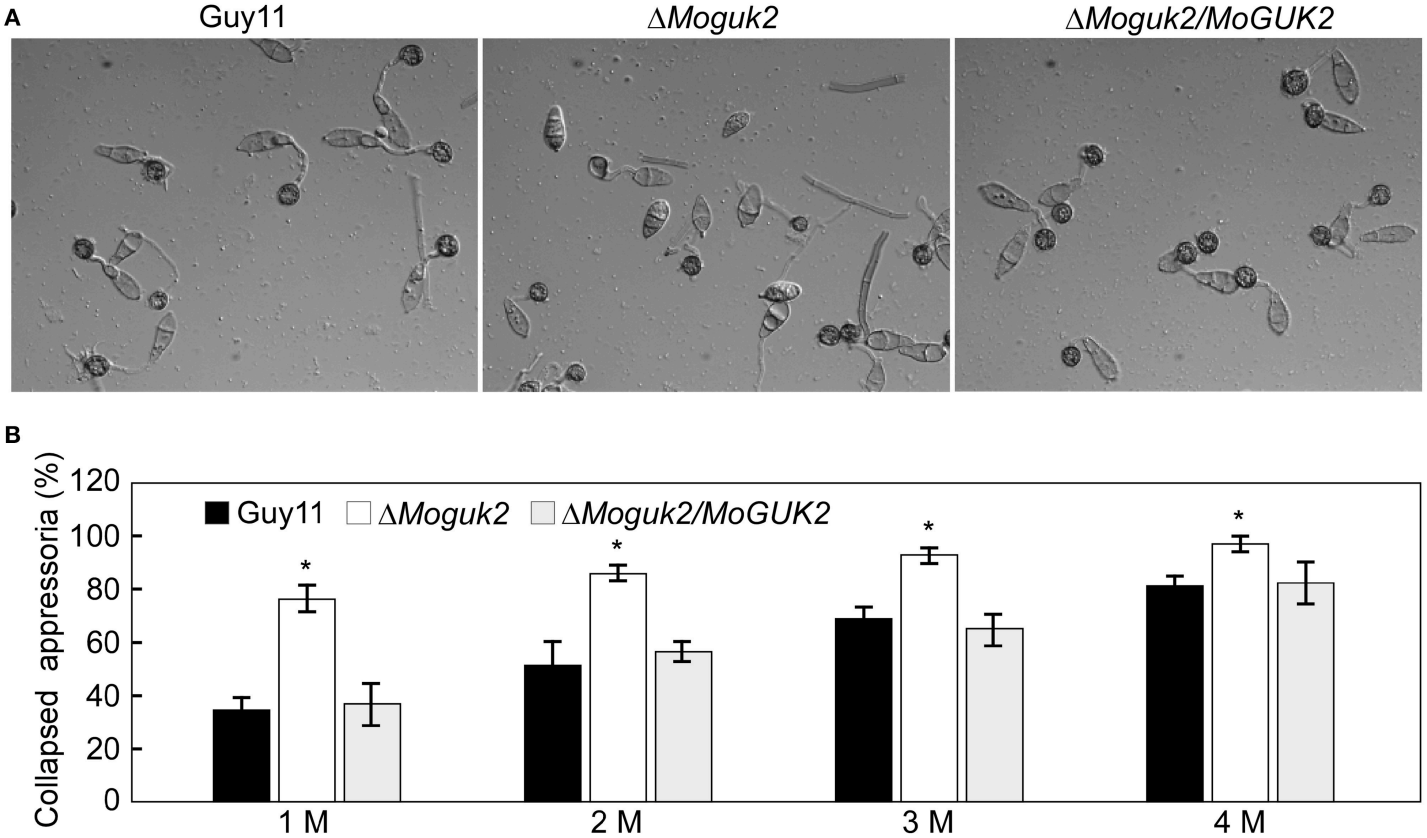
The Δ*Moguk2* mutant showed defects in appressorial formation and turgor. **(A)** Appressorial formation was observed on hydrophobic surfaces under a microscope at 24 hpi. **(B)** Appressorial turgor measurement. Error bars are standard deviations and asterisks represent significant differences at *P* < 0.01. All graphs represent three biological and three technical replicate observations.

Appressoria are specialized structures that facilitate penetration of the rice blast fungus (Saunders et al., 2010). Because the Δ*Moguk2* mutant caused only a few lesions on rice leaves, we speculated that the mutant might have defects in appressorial formation or penetration. To assess this possibility, we first observed conidial germination and appressorial formation on inductive surfaces. The results showed that both of these two processes were delayed by 6 h in the mutant compared to the wild type (Figure 6A, Table 2), indicating MoGuk2 plays a role in conidial germination and appresssorial formation in *M. oryzae*. We also measured the appressorial turgor, the mutant showed decreased turgor pressure compared with the wild type, suggestive of a defect in appressorial function (Figure 6B).

**Table 2:** Conidial germination and appressorial formation of the indicated strains with or without exogenous GDP or ATP.

**Strain**	**Conidial germination (%)**							**Appressorial formation (%)**						
	**4 h**	**6 h**	**8 h**	**10 h**	**12 h**	**24 h**	**48 h**	**4 h**	**6 h**	**8 h**	**10 h**	**12 h**	**24 h**	**48 h**
Guy11	90.7 ± 3.1A	96.7 ± 1.2A	99.4 ± 1.2A	100.0 ± 0A	100.0 ± 0A	100.0 ± 0A	100.0 ± 0*A*	74.7 ± 4.2A	90.0 ± 2.0A	100.0 ± 0A	100.0 ± 0A	100.0 ± 0A	100.0 ± 0A	100.0 ± 0A
Δ*Moguk2*	10.2 ± 3.51D	46.7 ± 3.11C	58.7 ± 3.1C	60.1 ± 2.4C	62.0 ± 3.4C	66.0 ± 4.0C	82.0 ± 2.0B	0	28.7 ± 4.2D	66.0 ± 2.0C	76.7 ± 2.3B	74.7 ± 3.7C	79.4 ± 3.11D	98.0 ± 2.0A
Δ*Moguk2*+GDP	62.0 ± 3.5B	82.7 ± 4.2B	86.0 ± 2.0B	92.7 ± 3.01A	94.7 ± 2.3A	97.4 ± 1.21A	100.0 ± 0A	1.7 ± 1.0C	36.7 ± 4.21D	66.7 ± 3.1C	75.3 ± 3.0B	78.0 ± 2.01C	94.7 ± 1.2B	100.0 ± 0A
Δ*Moguk2*+ATP	NA	NA	NA	NA	NA	97.0 ± 1.0A	100.0 ± 0A	NA	NA	NA	NA	NA	96.0 ± 1.0*AB*	100.0 ± 0A
MoGuk2^ΔefThoc1^	31.3 ± 2.3C	48.7 ± 3.1C	64.0 ± 4.01C	69.4 ± 6.1B	70.0 ± 2.0B	90.7 ± 3.1B	NA	0	48.0 ± 6.0C	66.0 ± 3.4C	74.6 ± 2.3B	86.7 ± 2.3B	90.0 ± 2.01C	NA
MoGuk2^ΔGuKc^	20.7 ± 4.2D	50.7 ± 3.11C	62.7 ± 3.11C	72.0 ± 4.0B	73.0 ± 3.01B	90.7 ± 2.31B	NA	0	30.7 ± 1.21D	64.0 ± 3.4C	70.6 ± 1.2B	86.7 ± 2.31B	88.7 ± 1.21C	NA
MoGuk2^ΔPL^	59.3 ± 4.2B	77.4 ± 4.2B	91.4 ± 2.4B	94.0 ± 2.01A	94.7 ± 2.3A	95.4 ± 1.21AB	NA	28.7 ± 4.2B	60.7 ± 1.2B	89.4 ± 2.3B	95.4 ± 3.11A	96.0 ± 2.01C	96.7 ± 1.21AB	NA
MoGuk2	89.5 ± 2.1A	94.8 ± 2.0A	98.9 ± 1.61A	100.0 ± 0A	100.0 ± 0A	100.0 ± 0A	NA	72.8 ± 3.6A	88.0 ± 3.21A	100.0 ± 0A	100.0 ± 0A	100.0 ± 0A	100.0 ± 0A	NA

*±SD was calculated from three repeated experiments and different letters represent significant differences at P < 0.01. NA, not assayed*.

### MoGuk2 plays a role in IH growth

**Figure 7:**
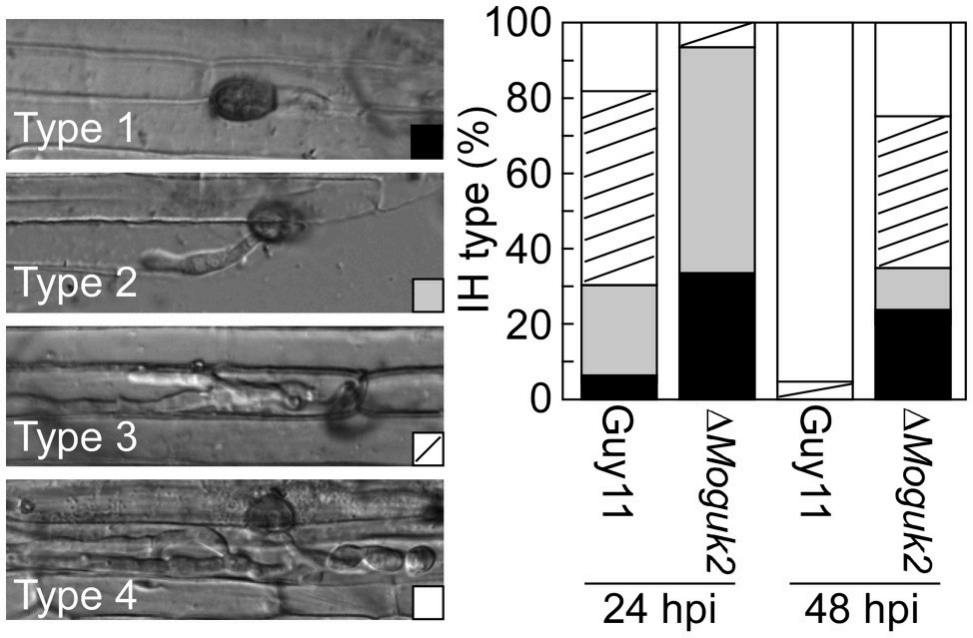
The Δ*Moguk2* mutant was defective in infectious hyphal growth. Statistical analysis of the percentage of infectious hyphae (IH) types in rice sheath cells at 24 and 48 hpi (type 1: no infectious hyphae; type 2: one infectious hyphae; type 3: two or three branches restricted in one cell; type 4: more than three branches extended to a neighboring cell; *N* = 100).

As described above, the Δ*Moguk2* mutant was defective in appressorial turgor and caused small lesions on rice seedlings, suggesting defects in penetration and IH growth. Therefore, penetration and IH growth of the mutant were examined in rice sheath cells. After incubation for 24 h, 33% of the appressoria of the mutant were unable to penetrate rice epidermal cells (type 1), 67% of IH were restricted to one cell with no (type 2) or two branches (type 3), and almost no IH extended to neighboring cells with three or more branches (type 4) In contrast, almost all appressoria penetrate into the cells and showed severe IH growth (Figure 7). When observed at 48 hpi, IH growth became severe in the mutant, percentage IH of type 3 and type 4 increased to 76%. (Figure 7), indicating that IH growth was delayed by 24 h in the Δ*Moguk2* mutant compared to the wild type.

### Exogenous GDP and ATP partially restored the defects of the Δ*Moguk2* mutant

**Figure 8:**
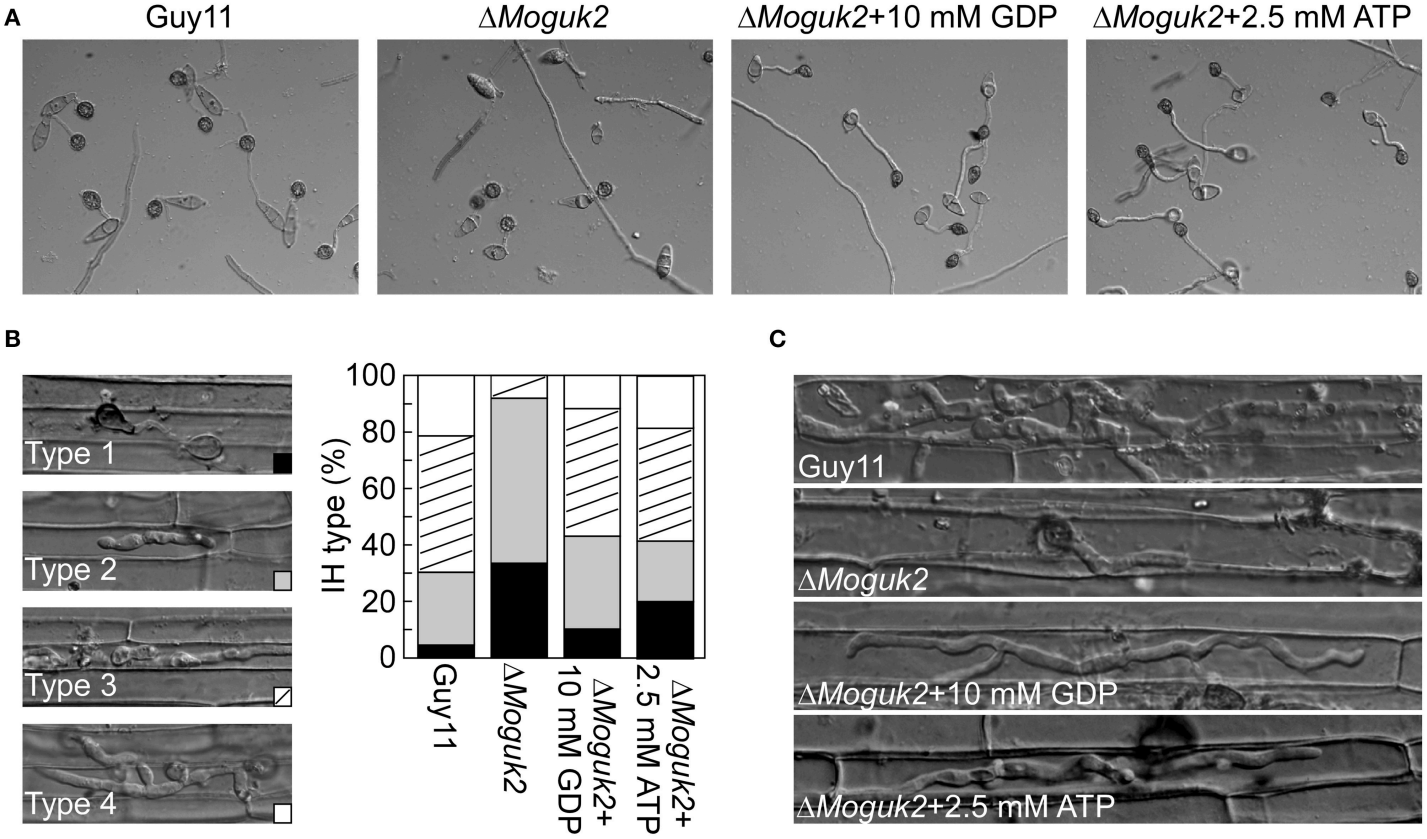
Exogenous GDP and ATP partially restored the defects of the Δ*Moguk2* mutant. **(A)** Appressorial formation of the Δ*Moguk2* mutant with or without exogenous GDP or ATP, and photographed at 24 hpi. **(B)** Statistical analysis the infectious hyphae (IH) type of the Δ*Moguk2* mutant in rice leaf sheath with or without exogenous GDP or ATP, and photographed at 30 hpi. **(C)** IH observation of the Δ*Moguk2* mutant with or without exogenous GDP or ATP at 40 hpi.

To determine whether MoGuk2 plays a role in *de novo* GTP biosynthesis, the Δ*Moguk2* mutant was treated with GDP, the immediate product of GK. Addition of 10 mM exogenous GDP restored conidial germination in the Δ*Moguk2* mutant (Table 2). Appressorial formation was also restored, albeit not to the wild type level, at 24 hpi (Figure 8A, Table 2). Observation of IH growth revealed that Δ*Moguk2*+GDP displayed longer IH with more branches (40% type 3 and 12% type 4), in comparison with 33% type 1 (no penetration) and 55% type 2 (with no branches) in the Δ*Moguk2* mutant (Figure 8B). At 40 hpi, the Δ*Moguk2* mutant showed short IH without branches, and Δ*Moguk2*+GDP showed longer IH, with one or two branches restricted to one plant cell. In contrast, the wild type Guy11 exhibited IH with many branches extending to neighboring cells (Figure 8C). We also treated the Δ*Moguk2* mutant with exogenous ATP, addition of 2.5 mM ATP restored the conidial germination, appressorial formation as well as infectious growth defects of the mutant (Figures 8A–C; Table 2). However, both GDP and ATP failed to restore vegetative growth, conidiation and conidial morphology of the mutant (data not shown). We also added exogenous GTP in the Δ*Moguk2* conidial suspensions to test the virulence on barley leaves, and found 2.5 mM GTP could restore the virulence defect of the mutant (Figure S3). These results indicate that MoGuk2 plays a role in the *de novo* GTP biosynthesis pathway in *M. oryzae*.

### The Δ*Moguk2* mutant is sensitive to neomycin and neomycin-tunicamycin

In yeast, guanylate kinase Guk1 mutants displayed a strong defect in protein *N*-glycosylation and cell wall organization that leads to a hypersensivity to neomycin, and a mixture of neomycin-tunicamycin (Shimma et al., 1997). To investigate whether MoGuk2 has a similar role in *M. oryzae*, the wild type Guy11, Δ*Moguk2* mutant and complemented transformant were inoculated onto CM agar plates with neomycin and neomycin-tunicamycin (an inhibitor of *N*-glycosylation) mixture. After 7 days incubation, the results showed that the mutant was more sensitive to the drugs compared to the wild type and complemented transformant. The growth rate was decreased 11.3 and 25.9% on neomycin, neomycin-tunicamycin, compared to 1.3 and 16.1% in the wild type, respectively (Table 1).

### The domains of MoGuk2 are essential for its full function

**Figure 9:**
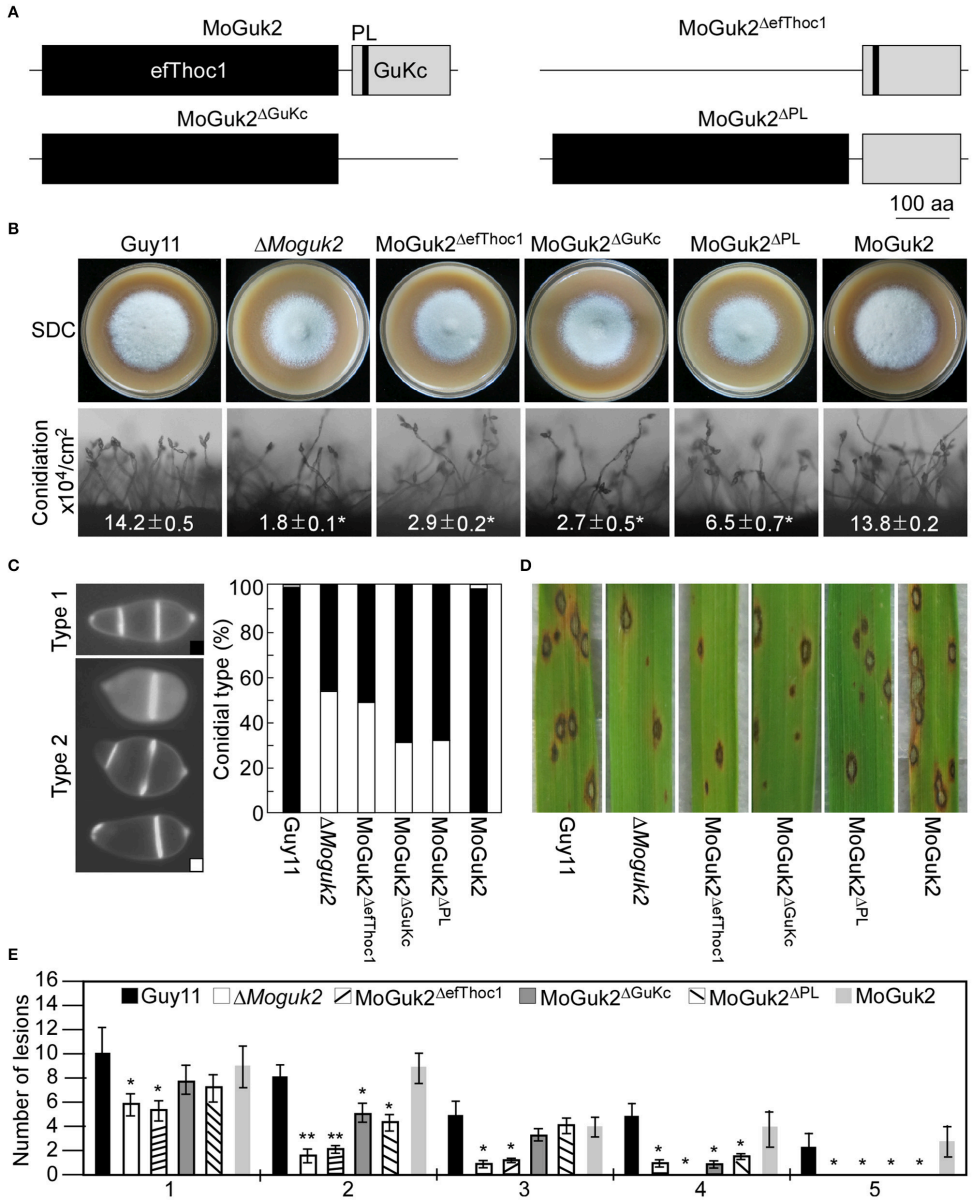
The efThoc1, GuKc, and P-loop domains are required for the full function of MoGuk2. **(A)** Domain deletion strategy and diagram of MoGuk2. **(B)** Vegetative growth and conidiation of the indicated strains. **(C)** Statistical analyzes the abnormal conidia of the indicated strains. **(D)** Spraying assay. Conidial suspensions prepared from the indicated strains were sprayed onto 2-week-old rice seedlings, and photographed at 7 dpi. **(E)** Statistical analysis of the number of lesions in each type. Error bars are standard deviations and asterisks represent significant differences at *P* < 0.01.

To investigate the contributions of its various domains to the function of MoGuk2, efThoc1, GuKc, and PL domain-deleted constructs (MoGuk2^ΔefThoc1^, MoGuk2^ΔGuKc^, and MoGuk2^ΔPL^, respectively, Figure 9A) were generated and transformed into the Δ*Moguk2* mutant. The resulting transformants were screened by quantitative RT-PCR (Figure S4). All three transformants showed similar phenotypes to that of the Δ*Moguk2* mutant, and only a few defects were partially restored. The vegetative growth of all three transformants was indistinguishable from that of the Δ*Moguk2* mutant (Figure 9B). Conidial production was partially rescued in the transformants, particularly in MoGuk2^ΔPL^ (Figure 9B). Conidial morphology and virulence were not rescued in MoGuk2^ΔefThoc1^ but were partially rescued in MoGuk2^ΔGuKc^ and MoGuk2^ΔPL^. Moreover, MoGuk2^ΔPL^ caused more severe lesions on rice leaves in comparison with MoGuk2^ΔGuKc^ (Figures 9C,D). Disease severity was also quantified by the lesion-type scoring assay. MoGuk2^ΔefThoc1^ showed similar lesion number and types to the Δ*Moguk2* mutant. While MoGuk2^ΔGuKc^ and MoGuk2^ΔPL^ displayed more type 2 and type 3 lesions compared to the mutant (Figure 9E). Conidial germination and appressorial formation were also partially rescued in the three transformants, to the greatest degree in MoGuk2^ΔPL^ (Table 2). These results indicate that the domains are critical for MoGuk2 functions in conidiogenesis, appressorial formation, and virulence. The efThoc1 and GuKc domains are more important than the PL domain of MoGuk2.

## Discussion

The GTP *de novo* biosynthesis and salvage pathways play critical roles in the growth, development and virulence of diverse organisms (Kirsch and Whitney, 1991; Rodriguez-Suarez et al., 2007; Jiang et al., 2010; Morrow et al., 2012). The key enzymes of these pathways are potential targets of anticryptococcal drugs (Morrow et al., 2012). In this study, we identified and characterized the yeast Guk1 ortholog MoGuk1 and a novel family of guanylate kinase MoGuk2 in *M. oryzae* that involved in GTP *de novo* biosynthesis pathway. Phylogenetic analysis revealed that both MoGuk1 and MoGuk2 and its orthologs were well conserved in different species, but Guk1 and Guk2 proteins clustered in different group, indicating MoGuk2 belongs to a novel family of GK proteins. MoGuk1 showed very similar structure to yeast Guk1 which is an essential gene in yeast, indicating MoGuk1 might also be essential for *M. oryzae*, since we failed to obtain a null mutant by screening over 1,000 transformants. However, further experiments such as performing transformation in the presence of GTP or to perform promoter replacement need to be conducted to confirm this hypothesis.

Deletion of MoGuk2 resulted in a reduced growth rate on different media, including nutrient-rich (CM) and minimal media, this is different from the case in *C. neoformans*, in which purine bases are required for growth of mutants in the purine biosynthesis pathway (Morrow et al., 2012). One explanation might be the intracellular GDP and GTP levels of the mutant could still maintain its vegetative growth, though GDP and GTP levels were decreased in the mutant. However, exogenous GDP and ATP partially restored the conidial germination, appressorial formation, and IH growth defects of the mutant, but not conidial production and morphology, suggesting that MoGuk2 plays a role in the *de novo* GTP biosynthesis pathway which important for conidial germination, appressorial formation, and IH growth. Nevertheless, conidial production and morphology alteration was not observed in the related mutants that similar to the Δ*Moguk2* mutant in these organisms. The possible explanation is that there is another GK (MoGuk1) that is functional in the Δ*Moguk2* mutant. Like in yeast, the Δ*Moguk2* mutant was hypersensitivity to the drugs neomycin and neomycin-tunicamycin, indicating MoGuk2 probably plays a role in protein *N*-glycosylation and cell wall organization.

Appressoria and their normal function are critical for *M. oryzae* penetration of host cells (Howard et al., 1991; Saunders et al., 2010). More than 20% of the mutant conidia were unable to germinate on an inductive surface even at 48 hpi, possibly due to a defect in sensing surface signals or cell viability. However, infectious growth in host cells was restricted. Although the mutant had defect in appressorial turgor, the mutant could not restore the virulence on wounded barley leaves (Figure S5A), indicating that the attenuation of the Δ*Moguk2* mutant mainly due to IH growth defect. In plants, the generation of reactive oxygen species (ROS) is regarded as one of the first responses to fungal invasion (Mellersh et al., 2002). For plant pathogens, ROS also play an important role and loss of the ability to eliminate intracellular ROS can result in functional defects (Egan et al., 2007). Our result showed that the Δ*Moguk2* mutant did not induce plant defense response and ROS accumulation (Figure S5B), indicating the IH growth defects was not due to plant defense. In *M. oryzae*, virulence defects of the mutants with deletions in amino acid or uridine biosynthesis genes—such as *STR3/MET6/MET13, ILV1/2/6, LYS2/20, CPA2/ARG, ADE1*, and *PYR5*, could be restored by exogenous amino acids or uridine (Wilson et al., 2012; Du et al., 2013; Fernandez et al., 2013; Yan et al., 2013; Chen et al., 2014; Zhang et al., 2014, 2015; Saint-Macary et al., 2015; Liu et al., 2016; Qi et al., 2016). Similar to these mutants, IH growth defects of the Δ*Moguk2* mutant could be restored by exogenous GDP and ATP, suggesting GTP biosynthesis pathway plays a critical role in IH growth in *M. oryzae*.

Structural analysis revealed that MoGuk2 contains an efThoc1, a GuKc, and a PL domain. Deletion of each domain proved that all three domains are essential for the full function of MoGuk2. The efThoc1 domain seemed to be more important than the GuKc and PL domains in terms of conidial morphology and virulence. Although the PL motif contributes to the function of Guk2, other functional motifs are also present in the GuKc domain; PL was found to play a less important role in conidiation, appressorial formation, and infection than was GuKc. These three domains are likely involved in the *de novo* GTP biosynthesis pathway, and they likely affect the development and virulence of *M. oryzae*. Domain deletion might affect protein conformation and decrease or abolish its activity, thereby affecting the function of MoGuk2. MoGuk2 is an atypical GK and play important roles in many developmental processes, such as vegetative growth, asexual/sexual development, conidial morphology and germination, appressorial formation, and IH growth which closely related to GDP or ATP. These results are consistent to the findings in other fungi that mutants defective in the GTP biosynthesis are autotrophic and could be rescued by guanine (Konrad, 1992; Rodriguez-Suarez et al., 2007; Morrow et al., 2012). MoGuk1 and MoGuk2 likely functional redundancy in some respects, such as GDP biosynthesis, but MoGuk1 seems is a more important protein, as it was likely essential for *M. oryzae*.

## Conclusion

This study identified and characterized an atypical GK MoGuk2, which plays a role in the *de novo* GTP biosynthesis pathway and is important for infection-related morphogenesis in the rice blast fungus. Our findings will improve our understanding of the pathogenic mechanisms of phytopathogens.

## Author contributions

XC, XZha, HZ, and ZZ: conceived and designed the experiments; XC, XZha, ML, XLi, XLiu, and XW: performed the experiments; XC, XZha, ML, XLiu, and HZ: analyzed the experiment data; XC, XZha, ML, XW, HZ, and XZhe: contributed reagents, materials, analysis tools; XC, XZha, HZ, and ZZ: wrote the paper; All authors have read and approve the final manuscript.

### Conflict of interest statement

The authors declare that the research was conducted in the absence of any commercial or financial relationships that could be construed as a potential conflict of interest.
