# Supplementary material for: The Atypical Guanylate Kinase MoGuk2 Plays Important Roles in Asexual/Sexual Development, Conidial Septation, and Pathogenicity in the Rice Blast Fungus
Source: Front Microbiol. 2017 Dec 11;8:2467. doi: 10.3389/fmicb.2017.02467 (PMC5732230; doi:10.3389/fmicb.2017.02467)
Supplement: Supplementary file 1 [file Table1.PDF]

Supplementary Material

The atypical guanylate kinase MoGuk2 plays important roles in asexual/sexual development, conidial septation and pathogenicity in the rice blast fungus

Xingjia Cai[$], Xi Zhang[$], Xinrui Li, Muxing Liu, Xiaoli Wang, Haifeng Zhang*, Xiaobo Zheng, and Zhengguang Zhang

*** Correspondence:** Haifeng Zhang: hfzhang@njau.edu.cn

1. Supplementary Table

Table S1. Primers used in this study.

Primer name	Sequence (5'-3')	Description
MGG_06764F1	CCCAAGCTTCCAAGAGCAGCTGTTCGTG	amplifiy *MoGUK1* 5' flank sequence
MGG_06764R2	CCGGAATTCGCCCCGCGTGCGGGATCTC	amplifiy *MoGUK1* 5' flank sequence
MGG_06764F3	GGACTAGTACAGGAGGGGGCCGCATCAGAT TAA	amplifiy *MoGUK1* 3' flank sequence
MGG_06764R4	TCCCCGCGGCCTAGCAGTTGCTAATCTCTGT	amplifiy *MoGUK1* 3' flank sequence
MGG_06764KOF	AGTCCAGCATATCAGCCCGCT	Screen *MoGUK1* transformants
MGG_06764KOR	ACTCGTCGAGTTCTTTGAACG	Screen *MoGUK1* transformants
MGG_06394F1	ACTCGAGCTCGAACAGAGCCCAGGTAT	amplifiy *MoGUK2* 5' flank sequence
MGG_06394R2	GATATCTGTTGCCAACTAATCCAAGA	amplifiy *MoGUK2* 5' flank sequence
MGG_06394F3	TCTAGAGTATGGGTATAGGAGGCAGA	amplifiy *MoGUK2* 3' flank sequence
MFF_06394R4	GAGCTCAAGTGTCCCTTCATACAGCT	amplifiy *MoGUK2* 3' flank sequence
MGG_06394GFPF	ACTCACTATAGGGCGAATTGGGTACTCAAATTGGTT TGGCGGTAGAAATAGAAGA	amplify *MoGUK2* complementation sequence
MGG_06394GFPR	CACCACCCCGGTGAACAGCTCCTCGCCCTTGCTCAC CTCCTGCTCCTGGGCCTGCT	amplify *MoGUK2* complementation sequence
FL1111	GGAGGTCAACACATCAATG	amplify *HPH* probe
FL1112	CTCTATTCCTTTGCCCTCG	amplify *HPH* probe
MGG_06394KOF	CACGTGGAGAATGTCACAAC	amplify *MoGUK2* probe

MGG_06394KOR	GAATATATCTCCGGAAGGTG	amplify *MoGUK2* probe
F-EF-UP	ACTCACTATAGGGCGAATTGGGTACTCAAATTGGTT ATCCTGTAGCAGGGTGTT	MoGuk2 efThoc1 domain deletion
R-EF-UP	CAACGGCCGCAAGTCCCTGACAAAATCCGG	MoGuk2 efThoc1 domain deletion
F-EF-DOWN	CCGGATTTTGTCAGGGACTTGCGGCCGTTG	MoGuk2 efThoc1 domain deletion
R-EF-DOWN	CAC CAC CCC GGT GAA CAG CTC CTC GCC CTT GCT CACGCTTAGTCCCGCCCAT	MoGuk2 efThoc1 domain deletion
F-GuKc	ACTCACTATAGGGCGAATTGGGTACTCAAATTGGTT ATCCTGTAGCAGGGTGTT	MoGuk2 GuKc domain deletion
R- GuKc-1	ATCTGGCTGCTTTCCTGG	MoGuk2 GuKc domain deletion
R- GuKc-2	CACCACCCCGGTGAACAGCTCCTCGCCCTTGCTCAC GCTTAGTCCCGCCCATGGTCTCGTCTTCCCCAGACG TATCTGGCTGCTTTCCTGG	MoGuk2 GuKc domain deletion
F-PL-DOWN	ACTCACTATAGGGCGAATTGGGTACTCAAATTGGTT ATCCTGTAGCAGGGTGTT	MoGuk2 P-loop domain deletion
R-PL-DOWN	AGGGTTCCGTTCTTGCGGCCGCAAGTCAT	MoGuk2 P-loop domain deletion
F-PL-DOWN	ATGACTTGCGGCCGCAAGAACGGAACCCT	MoGuk2 P-loop domain deletion
R-PL-DOWN	CACCACCCCGGTGAACAGCTCCTCGCCCTTGCTCAC GCTTAGTCCCGCCCAT	MoGuk2 P-loop domain deletion
06764-DL-F	TTCCAGGCCCTGATTGCC	Quantitative PCR analysis of *MoGUK1*
06764-DL-R	ACTCGTCGAGTTCTTTGAACG	Quantitative PCR analysis of *MoGUK1*
06394-DL-F	ATGCCTTTACTTGACATTGACA	Quantitative PCR analysis of *MoGUK2*
06394-DL-R	TGGTTGCGCAGTATCTG	Quantitative PCR analysis of *MoGUK2*
06764PYES2-F	CCGGGATCCATGTCGAAACTGGGATT	amplify *MoGUK1* cDNA
06764PYES2-R	CGCGAATTCCTACTGTGCCGGCATTTCGT	amplify *MoGUK1* cDNA
06394PYES-F	AAGCTTGGTACCGAGCTCGGATGCCTTTACTTGACA TT	amplify *MoGUK2* cDNA
06394PYES-R	TGATGGATATCTGCAGAATTTCAGCTTAGTCCCGCC CAT	amplify *MoGUK2* cDNA
