# Supplementary material for: The Atypical Guanylate Kinase MoGuk2 Plays Important Roles in Asexual/Sexual Development, Conidial Septation, and Pathogenicity in the Rice Blast Fungus
Source: Front Microbiol. 2017 Dec 11;8:2467. doi: 10.3389/fmicb.2017.02467 (PMC5732230; doi:10.3389/fmicb.2017.02467)
Supplement: Supplementary file 2 [file Image1.PDF]

Supplementary Material

The atypical guanylate kinase MoGuk2 plays important roles in asexual/sexual development, conidial septation and pathogenicity in the rice blast fungus

Xingjia Cai[$], Xi Zhang[$], Xinrui Li, Muxing Liu, Xiaoli Wang, Haifeng Zhang*, Xiaobo Zheng, and Zhengguang Zhang

*** Correspondence:** Haifeng Zhang: hfzhang@njau.edu.cn

1. Supplementary Figure

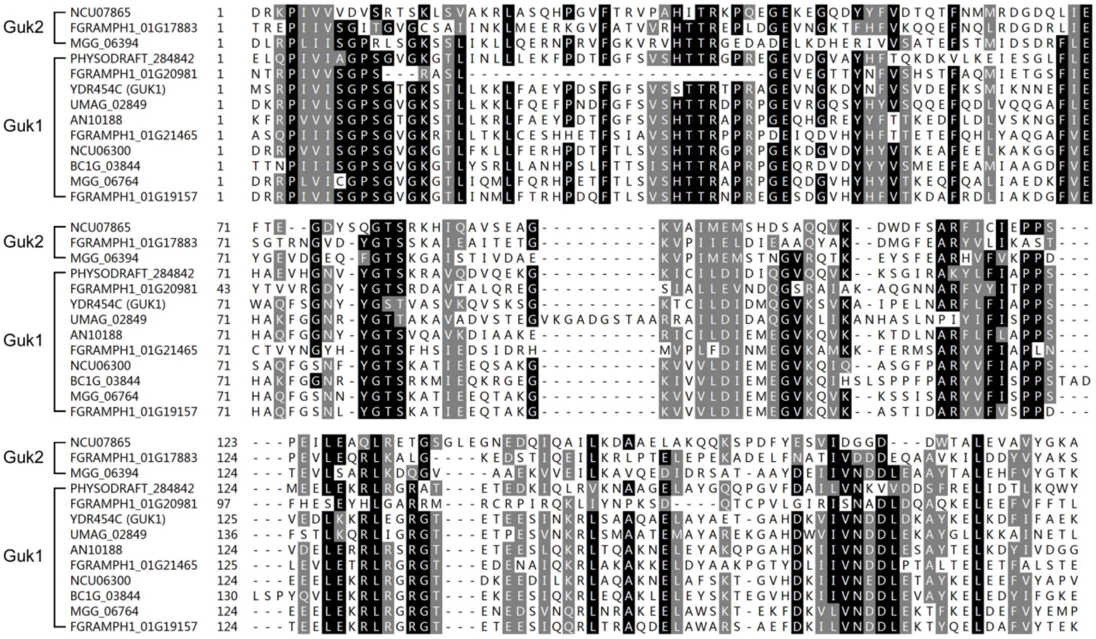

Supplementary Figure 1. Alignment of amino acid sequences of GuKc domain from different organisms. Identical and similar residues are shaded in black and gray, respectively.
