# Supplementary material for: The Atypical Guanylate Kinase MoGuk2 Plays Important Roles in Asexual/Sexual Development, Conidial Septation, and Pathogenicity in the Rice Blast Fungus
Source: Front Microbiol. 2017 Dec 11;8:2467. doi: 10.3389/fmicb.2017.02467 (PMC5732230; doi:10.3389/fmicb.2017.02467)
Supplement: Supplementary file 3 [file Image2.PDF]

Supplementary Material

The atypical guanylate kinase MoGuk2 plays important roles in asexual/sexual development, conidial septation and pathogenicity in the rice blast fungus

Xingjia Cai[$], Xi Zhang[$], Xinrui Li, Muxing Liu, Xiaoli Wang, Haifeng Zhang*, Xiaobo Zheng, and Zhengguang Zhang

*** Correspondence:** Haifeng Zhang: hfzhang@njau.edu.cn

1. Supplementary Figure

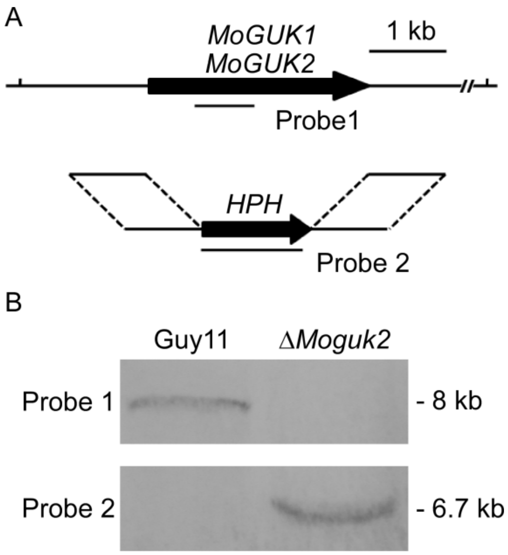

Supplementary Figure 2. Targeted deletion of *MoGUK1* and *MoGUK2* in *M. oryzae*. (A) Diagram of *MoGUK1* and *MoGUK2* deletion strategy in *M. oryzae* genome. (B) Southern blot analyzes the *MoGUK2* gene deletion mutant.
