# Supplementary material for: The Atypical Guanylate Kinase MoGuk2 Plays Important Roles in Asexual/Sexual Development, Conidial Septation, and Pathogenicity in the Rice Blast Fungus
Source: Front Microbiol. 2017 Dec 11;8:2467. doi: 10.3389/fmicb.2017.02467 (PMC5732230; doi:10.3389/fmicb.2017.02467)
Supplement: Supplementary file 4 [file Image3.PDF]

Supplementary Material

The atypical guanylate kinase MoGuk2 plays important roles in asexual/sexual development, conidial septation and pathogenicity in the rice blast fungus

Xingjia Cai[$], Xi Zhang[$], Xinrui Li, Muxing Liu, Xiaoli Wang, Haifeng Zhang*, Xiaobo Zheng, and Zhengguang Zhang

*** Correspondence:** Haifeng Zhang: hfzhang@njau.edu.cn

1. Supplementary Figure

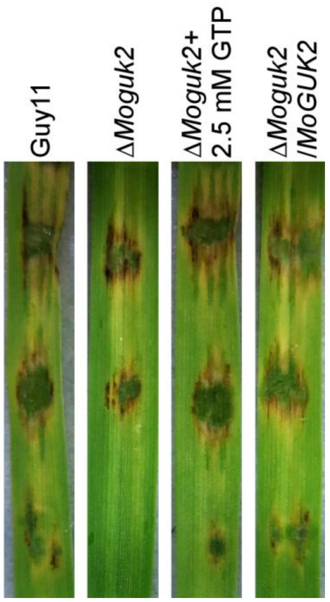

Supplementary Figure 3. Exogenous GTP could restore the virulence defect of the Δ*Moguk2* mutant on barley leaves. Conidial suspensions (1×10^5, 1×10^4 and 1×10^3 spores/ml) from the indicated strains with 2.5 mM GTP were dropped onto one-week-old detached barley leaves, and photographed at 5 dpi.
